# Developing a workbook to support the contextualisation of global health systems guidance: a case study identifying steps and critical factors for success in this process at WHO

**DOI:** 10.1186/s12961-018-0297-x

**Published:** 2018-03-02

**Authors:** Elizabeth Alvarez, John N. Lavis, Melissa Brouwers, Lisa Schwartz

**Affiliations:** 1 Department of Health Research Methods, Evidence and Impact (HEI), McMaster University, CRL 2nd Floor, 1280 Main Street West, Hamilton, ON L8S 4K1 Canada; 2 McMaster Health Forum, MML-417, 1280 Main St. West, Hamilton, ON L8S 4L6 Canada; 3 Department of Oncology, McMaster University, Juravinski Hospital Site G Wing, 2nd Floor, Room 207, 711 Concession Street, Hamilton, ON L8V 1C3 Canada

**Keywords:** Guidance, Contextualisation, Knowledge translation, Health system strengthening, Political system, Qualitative research, Case study

## Abstract

**Background:**

Global guidance can help countries strengthen their health systems to deliver effective interventions to their populations. However, to have an impact, guidance needs to be contextualised or adapted to local settings; this process includes consideration of health system arrangements and political system factors. To date, methods to support contextualisation do not exist. In response, a workbook was designed to provide specific methods and strategies to enable the contextualisation of WHO’s ‘Optimizing health worker roles to improve maternal and newborn health’ (OptimizeMNH) guidance at the national or subnational level. The objective of this study was to describe the process of developing the workbook and identify key steps of the development process, barriers that arose and facilitators that helped overcome some of these barriers.

**Methods:**

A qualitative single case study design was carried out. Interviews, documents and a reflexive journal were used. Constant comparison and an edit-style of organisation were used during data analysis to develop concepts, themes, subthemes and relationships among them.

**Results:**

Thirteen interviews were conducted and 52 documents were reviewed. Three main steps were identified in the process of developing the workbook for health systems guidance contextualisation, namely (1) determining the need for and gaining approval to develop the workbook, (2) developing the workbook (taking on the task, creating the structure of the workbook, operationalising its components, undergoing approval processes and editing it), and (3) implementing the workbook both at the WHO level and at the national/subnational level.

Five barriers and/or facilitators emerged relevant to each step, namely (1) having well-placed and credible champions, (2) creating and capitalising on opportunities, (3) finding the right language to engage various actors and obtain buy-in, (4) obtaining and maintaining meaningful buy-in, and (5) ensuring access to resources.

**Conclusions:**

Understanding the key steps and the critical factors involved in the process of developing the workbook could help in the planning of similar and other tools aimed to support the implementation of WHO guidance. A plan for dissemination and implementation needs to be addressed during the preparation of these tools.

**Electronic supplementary material:**

The online version of this article (10.1186/s12961-018-0297-x) contains supplementary material, which is available to authorized users.

## Background

Many low-resource and effective clinical and public health interventions (e.g. immunisations, kangaroo mother care for low birthweight infants) are not reaching people who need them most, especially in low- and middle-income countries (LMICs), because of fragmented and overburdened health systems [1–4]. This also leads to the inability to implement evidence-based guideline recommendations. ‘Health systems guidance’, defined as “*systematically developed statements produced at global or national levels to assist decisions about appropriate options for addressing a health systems challenge in a range of settings*” [1], can be used to strengthen health systems by addressing issues such as the lack of trained health workers for the delivery of effective interventions. WHO is the leading producer of health systems guidance to help countries that are facing the same or similar system-level issues. Global guidance can be used to develop policies at the global level (e.g. funding policies of international organisations) and guidance or policies at the national or subnational levels (e.g. health human resources planning for HIV treatment) [5].

However, the process of using this evidence-based guidance is complex. In order to have an impact, the topic of the guidance needs to get on the government’s agenda, guidance has to inform policy development, and the policy needs to be implemented [5]. These steps are determined by whether a government agrees to prioritise a particular framing of a problem and its causes, whether it agrees that the recommendations make sense for its health system and in the context of its political system, and whether it has the commitment and resources to implement it [1, 5, 6]. Therefore, to increase the relevance, acceptance and implementation of global guidance, it needs to be contextualised or adapted to fit a particular setting [5]. Indeed, there are a few striking examples where the context has been noted to shape the implementation of guidelines [7–10]. For example, Bollini et al. [7] showed that the content of policies around HIV prevention in prisons in four different settings varied and reflected the thinking and strategies of HIV prevention and care in their respective communities despite the availability of international guidelines on this topic.

There has also been a lack of support for users of health systems guidance (i.e. policy-makers, stakeholders and researchers) for combining global recommendations with national/subnational assessments of local problems and their causes, as well as of existing health system arrangements that may need to be changed and political system considerations that need to be taken into account [5]. Part of this challenge has been noted to include a lack of implementation plans in WHO guidelines or specific strategies where WHO recommendations can be appropriately and systematically tailored for each jurisdiction [11, 12].

To address these challenges, the development of a workbook was proposed to help users of WHO recommendations be able to contextualise recommendations to their own setting or situation, with the ultimate goal of increasing the acceptability and implementability of the recommendations. This study describes the process of how this tool was developed, including the steps in the process and the critical factors leading to success or non-success in each step. An edited version of the workbook can be found at the McMaster Health Forum website at https://www.mcmasterforum.org/lets-collaborate/resources [13].

## Methods

### Context for this study

Between 2010 and 2012, WHO developed a guidance document with recommendations for optimising health workers’ roles (through regulation, training and support) to improve access to and utilisation of key interventions for improving maternal and newborn health in LMICs (OptimizeMNH guidance) [14]. The development of these guidelines is outside the scope of this paper, but was performed following WHO Guideline Review Committee processes and can be found at http://optimizemnh.org/. During the development of the guideline, it was recognised that tailoring recommendations to the health system context and political environment (e.g. agreement on the need for regulatory change for health cadres) (personal correspondence, Alvarez) was required to optimise their implementation. In response, the guidance developers requested a tool or resource to advise users on how to contextualise global recommendations to the unique situations in specific jurisdictions. A workbook for contextualising health systems guidance (henceforth ‘workbook’) was developed de novo by two of the authors of this paper (EA and JL). This study was pragmatic in that there was an opportunity to study the course of the development of the workbook while it occurred.

#### Framework underpinning the workbook

The workbook was based on scholarly and applied work in the field of health systems and policy [5, 15–28] and addressed (1) clarifying the problem and its causes, (2) framing options for addressing the problem, (3) identifying implementation considerations, (4) considering the broader health system context, (5) considering the broader political system context, (6) refining the statement of the problem, options and implementation considerations in light of health system and political system factors, (7) anticipating monitoring and evaluation needs and, finally, (8) making national or subnational policy recommendations or decisions [29]. These steps made up the ‘health systems guidance contextualisation framework’, which was created with the workbook.

### Study approach

Given the diversity of people developing health systems guidance within an international health organisation such as WHO, and given that this was a new process within WHO, an exploratory holistic single case design was chosen to allow analysis of the case and its context. This study design captures comprehensive and meaningful characteristics of complex real-life social phenomena [30]. Because multiple people were involved in the development of this workbook, a constructivist paradigm, where multiple realities are expected based on each person’s view, was fitting for this study [30, 31].

**Fig. 1:**
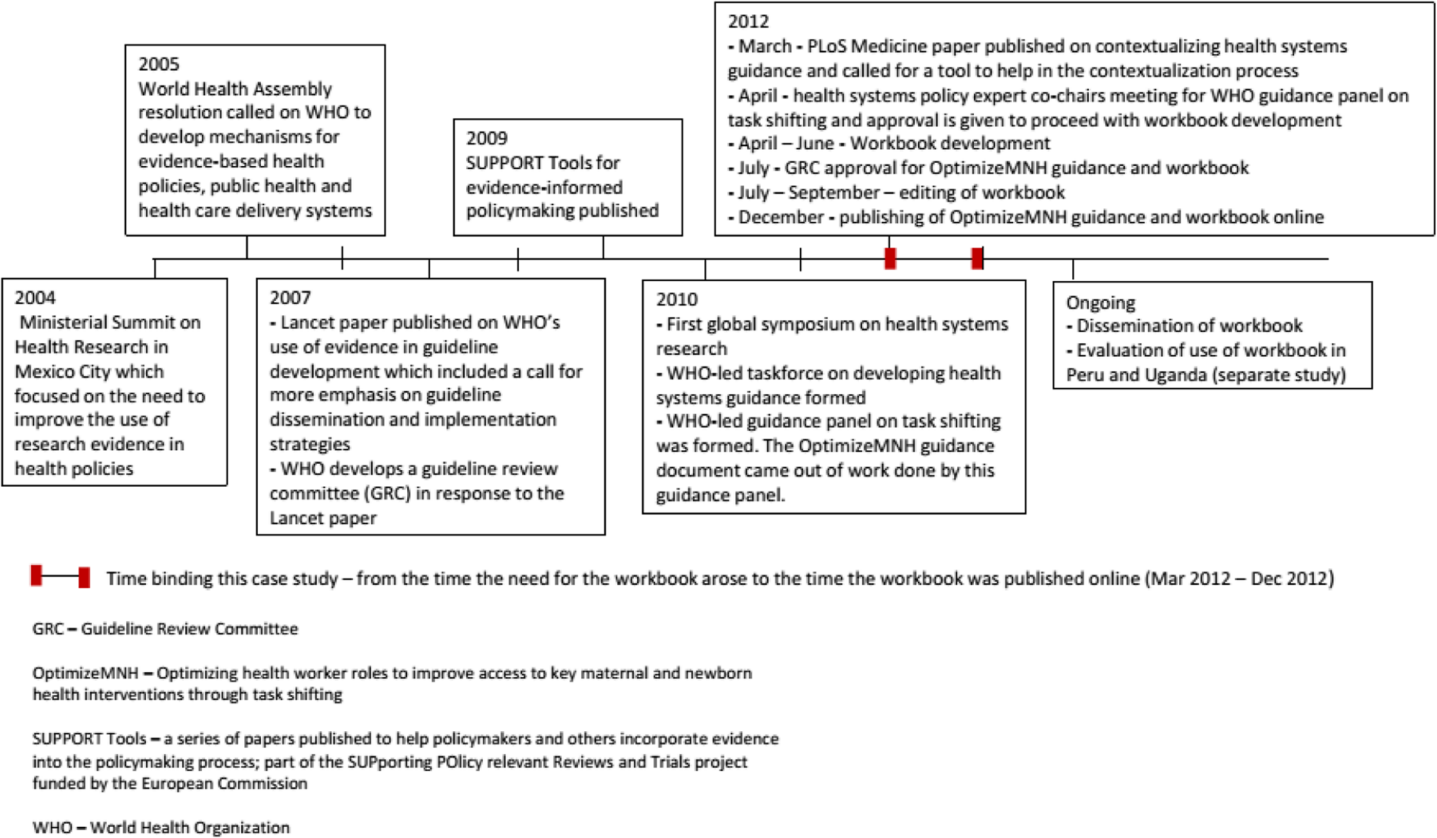
Timeline of events in the development of the workbook for contextualising health systems guidance

The ‘case’ in this investigation was the process of developing a workbook for the contextualisation of global health systems guidance. For the study to remain focused [30], it was bounded by context, so only information relating to the creation of the workbook was used; participants who took part in the process of developing the workbook; and time, starting when the workbook was conceptualised and ending when the workbook was posted online along with the OptimizeMNH guidance document (Fig. 1). WHO guidance documents, which would include the workbook, incorporate a continuous review process and therefore it was important to set a time limit for this study as changes could be ongoing for many years.

### Sampling and recruitment

Intensity sampling [32] was used to find participants who could provide in-depth information about the case, given that they were involved in the idea for or the actual design of the workbook. Respondent-driven sampling was also used to find other policy-makers, stakeholders or researchers who were considered to be information-rich sources about the case. Recruitment was conducted through personalised emails.

Criterion sampling [32] was used to find documents that related specifically to the process of developing the workbook, for example, meeting minutes. This was especially relevant because the development of the workbook was occurring in parallel with the development of the OptimizeMNH guidance, and some people were involved in both. These documents were found through literature searches, personal emails or interviews.

### Data collection

Informed consent was obtained prior to conducting the interviews. Semi-structured interviews were conducted by a single interviewer (EA) over Skype, and these were recorded with a digital recorder and transcribed. The interviewer asked participants about their role in, and the process of, the development of the workbook, challenges that arose during the process and how these challenges were overcome, other contributors to this work, and other documents for review. In addition, participants were asked to describe what their hopes were for a tool to help countries contextualise global guidance and the challenges they foresaw with such a tool.

Documents came from the interviews, from personal emails or through online searches by the principal investigator, and most were publicly available (Additional file 1). Finally, a reflexive journal was kept by the principal investigator (EA) throughout the course of the study to keep track of events and their contexts (e.g. who was involved, what and why decisions were made, etc.), to provide insights into the role of the principal investigator as a participant-observer, and to allow for personal reflection of the events [31]. Entries in this journal were also used as data.

### Data analysis

The coding of data was mainly conducted by the principal investigator (EA), with input from JL on the codes, themes and subthemes, and on the presentation of the data. Data analysis occurred concurrently with data collection to help direct further data collection. A code structure was developed using an integrated approach, where both deductive and inductive methods were used [33, 34]. A deductive organising framework or ‘start list’ [33, 34] was used, which included steps in the process of developing the workbook, challenges and mitigating strategies. Additional steps and sub-steps, challenges and mitigating strategies were added to the start list in a temporal sequence following an edit-style organising process using Microsoft Word [30, 34]. As this code structure was being developed, concepts, themes, subthemes and their relationships emerged inductively through constant comparison, where new information was evaluated against previous data. These concepts and themes were then verified across each main step in the process. The code structure and interview transcripts were reviewed to ensure no new themes or disconfirming evidence were found. Data saturation was achieved. Member checking with two interviewees and peer debriefing helped refine the concepts and their presentation.

## Results

### Participants

Overall, 13 of the 17 individuals approached participated in the study (76% response rate); 3 did not respond to the invitation and 1 was not persuaded they could make a meaningful contribution. For confidentiality purposes, identifying information is not presented, but there was diversity in age, sex, seniority and roles, and the participants had collective experience at the district, national and international levels as well as representing every WHO region except for South-East Asia. Broadly, for the purposes of this study, participants are described as belonging to one of three categories, namely members of the Secretariat of the guidance panel on task shifting (those involved in developing the WHO OptimizeMNH guidance who either worked at WHO or at an outside institution – 3 participants), staff of WHO not part of the Secretariat of the guidance panel on task shifting (3 participants), or health system and policy analysts (7 participants).

One interview was conducted with each participant. These interviews ranged from 19 to 65 min in length, with an average duration of 38 min.

### Documents

A total of 52 documents were reviewed, including 27 journal articles, 7 presentations and 18 other documents (e.g. meeting agendas, reports, etc.). Multiple personal emails and a reflexive journal (Volume I – 195 pages, volume II – 127 pages, and volume III – 93 pages) were also used as data (Additional file 1).

### Process

Three main steps, and various sub-steps, were identified in the process of developing the workbook for health systems guidance contextualisation (Fig. 1). These were (1) determining the need for and gaining approval to develop the workbook, (2) developing the workbook (taking on the task, creating the structure of the workbook, operationalising its components, undergoing approval processes and editing it), and (3) implementing the workbook both at the WHO level and at the national/subnational level.

The first step helped explain the purpose of the workbook and gave a concrete reason for the workbook to be developed, which was to contextualise the OptimizeMNH guidance. The second step ranged from the time approval was gained to move ahead with this work to the time the workbook was published online by WHO. The third step continues and encompasses the dissemination, implementation and institutionalisation of the use of the workbook both at the level of WHO and at the national/subnational level. This last phase is critical for improving the workbook itself through user testing but also for helping improve the uptake of health systems guidance recommendations, which is the purpose of the workbook. While uptake has not been universal, the workbook has been applied and evaluated in Peru and Uganda (in review).

### Critical factors affecting the development of the workbook

**Fig. 2:**
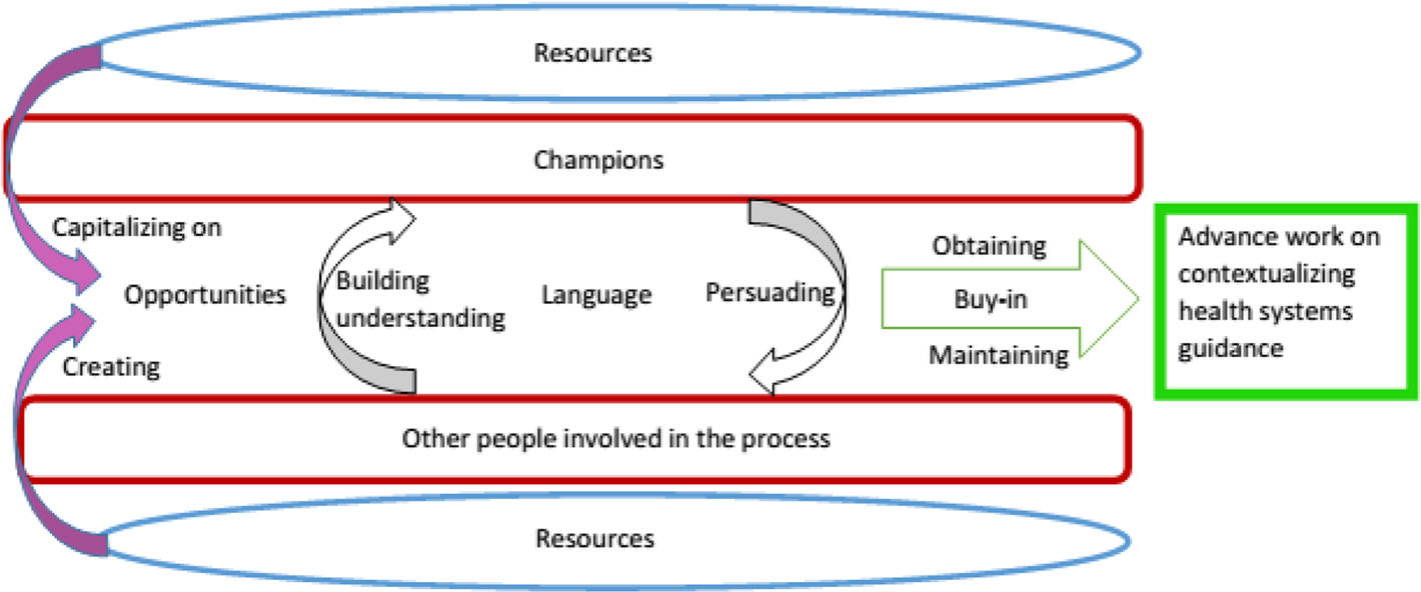
Relationships among critical factors influencing the development of the workbook for contextualising health systems guidance

At each of these three steps, five critical factors surfaced from the data as barriers and/or facilitators, including (1) having well-placed and credible champions, (2) creating and capitalising on opportunities, (3) finding the right language to engage various actors and obtain buy-in, (4) obtaining and maintaining meaningful buy-in, and (5) ensuring access to resources. General descriptions of each of these factors, along with their relationships, are provided (Fig. 2, Table 1). The implications of the presence or absence of these critical factors at each step are shown on Additional file 2, and a full description can be found elsewhere [35].

**Table 1:** Themes, subthemes and descriptions of critical factors influencing the process of workbook development

Themes	Subthemes	Descriptions of themes and subthemes
Having well-placed and credible champions	- People promoting ideas (Ideas) - People devoting their time/resources to completing the work (Work)	Champions were people who helped move ideas forward and/or who devoted their time and other resources to complete the work. Without their commitment and persistence, the work may not have occurred. Champions could have different levels of involvement, and someone who is a champion at one stage of the process may not be a champion at another.
Creating and capitalising on opportunities	- Creating opportunities (Creating) - Capitalising on opportunities (Capitalising) - Missed opportunities (Missed) - Potential opportunities (Potential)	The champion(s) either sought out or created opportunities to move the work forward, or other actors presented opportunities to the champion(s) who then capitalised on these opportunities. There were times when the work could have moved forward, but it did not, resulting in missed opportunities. Lastly, there are potential opportunities that could be capitalised upon to move the work even further in the future.
Finding the right language to engage various actors and obtain buy-in	- Building understanding by using common terminology (Building understanding) - Using persuasive language to ‘sell’ an idea (Persuading) - Following standards (e.g. use of scientific language, page length for publishing) (Standardising)	The champion(s) used persuasive language to gain buy-in from those who were involved in and could have created barriers in the process. However, a more subtle way to help obtain buy-in was to first build a shared understanding of the concepts or issues by using common terminology that resonated with the target audience. This level of shared understanding was seen as especially useful for trying to communicate the relevance of or how to apply the work. Following standards for language could be a facilitator when working with specific audiences, such as WHO’s Guideline Review Committee, which requires specific formatting and the use of academic language, but this can be a barrier to other audiences, such as policy-makers, who may not be familiar with scientific or academic language. Poor communication could act as a barrier.
Obtaining and maintaining meaningful buy-in	- Obtaining buy-in (Obtaining) - Maintaining buy-in for the current work (Maintaining) - Institutionalising the process or work (Institutionalising)	First, one needed to obtain buy-in and then buy-in needed to be maintained. Institutionalisation can be seen as a type of ‘permanent buy-in’ by an institution. Unless a process is institutionalised, a change in leadership could result in previous buy-in being lost. Therefore, either continuous communication with new leadership is required to secure meaningful buy-in from new leadership, or institutionalising a process by one group or leader could bypass the future need to obtain buy-in. The difficult part can be knowing if there is meaningful buy-in or if the buy-in is for a secondary purpose (e.g. advancing other work). This could manifest itself as appearing to have meaningful buy-in at one stage of the process but not having buy-in at another. However, ascertaining this level of information could require immense transparency on the part of the actors involved as it seems it would be unlikely for people to be open about secondary motives. Also, this problem could be difficult to distinguish from a separate problem of lacking resources (e.g. buy-in from one group of people may not secure resources from other groups of people).
Ensuring access to human, financial and other resources	- (Human resources) - (Motivation) - (Knowledge) - (Finances) - (Time) - (Technology)	Resources were used in carrying out the work and included human resources, finances, time, motivation, knowledge and technology. Without these resources, the work was likely to be abandoned. Human resources include the people involved in doing the work. Further attributes of these individuals, which arose from the data, include motivation and knowledge. Motivation to work on a particular topic is necessary when there are competing demands on an individual or on an agency. This can be seen as prioritising specific work. Knowledge can come from existing knowledge of the individuals involved in the work or can be found through searches for information. So, this attribute can be intrinsic (e.g. expertise) or extrinsic (e.g. library resources). Finances include salaries or payments for those carrying out the work, funding for traveling and funding to secure supplies. Time is required for individuals to do the work, and individuals and agencies have timelines for getting the work done. Technology can also support or be a barrier in advancing the work.

#### Having well-placed and credible champions

Through the interviews and reflexive journal, several people emerged as being more prominent in moving ideas forward and/or devoting their time and other resources to completing tasks in the process. Without these champions, the workbook may not have come to fruition. While champions may be thought of as people with clout or within existing leadership positions, our definition sees champions as being leaders from any position, but does require that they go beyond their day-to-day tasks in moving the ideas forward [36]. Champions can have different levels of involvement, and someone who is a champion at one stage of the process may not be a champion at another.

“*…so I think my role was more kind of being the person pushing for this to be brought about, identifying the opportunity for* [the person taking on this work as participant-observer]*, and then after that I moved into much more of a supporting role.*” (001, Health systems and policy analyst)

“*It took* [the head of the Secretariat of the WHO guidance panel on task shifting] *a lot of work in the background in the WHO to try and bring everyone on board* [to using innovations in the guidance development process]*.*” (002, Member of Secretariat of WHO guidance panel on task shifting)

#### Creating and capitalising on opportunities

The champion(s) either sought out or created opportunities to move the work forward, or other actors presented opportunities to the champion(s) who then capitalised on these opportunities.

“*We knew that the recommendations from the guidance were going to be directed to policy-makers and that is why we involved* [the health systems policy expert] *in the panel.*” (003, Member of Secretariat of WHO guidance panel on task shifting)

There were times when the work could have moved forward, but it did not, resulting in missed opportunities. One example is that there were several opportunities to promote the workbook to attendees at a WHO-African Regional Office meeting in Addis Ababa, Ethiopia, in 2012. However, a prior champion of this work did not bring up the potential to use and evaluate the workbook at the country level as was expected, creating missed opportunities. Lastly, there was a potential opportunity identified by a participant that could be capitalised upon in the future, involving identifying gaps in the evidence through the contextualisation process, which could then be fed back into and inform research processes.

#### Finding the right language to engage various actors and obtain buy-in

Compiling information from the interviews and journal, the concept surfaced that the champion(s) used persuasive language to gain buy-in from those who were involved in the process and who could have created barriers. A more subtle way to help obtain buy-in was to first build a shared understanding of the concepts or issues about contextualisation by using common terminology that resonated with the target audience. This level of shared understanding about what is meant by contextualisation and its importance was seen as especially useful for trying to communicate the relevance of the work or how to apply the work.

“*...he* [the head of the Secretariat of the WHO guidance panel on task shifting] *gave me the opportunity to pitch the idea to the committee members, which I did in the form of a brief presentation and they seemed to be quite excited about the idea. And I also, because in sharing the meeting…after two days, I was able to insert a bunch of examples that had come back, come directly from them, that were the type of things that a good workbook would flag for people*.” (001, Health systems and policy analyst)

Communication challenges within WHO were noted as a barrier, especially for disseminating the use of the workbook, including departments working in silos and products developed in one department not necessarily being communicated for use by other departments or at the regional office level through internal means. In addition, several participants stated that following standard academic or scientific language could be a facilitator when working with specific audiences, such as WHO’s Guideline Review Committee, but this could be a barrier for other audiences, such as policy-makers, who may not be familiar with this language. Likewise, the workbook had to be approved by the Guideline Review Committee and had to follow an academic style. Evaluating the use of the workbook will help determine if the workbook is easy to understand by research and lay audiences who will be using the workbook directly.

“*I think one of the criticisms we have had of the Optimize guidelines is that it feels, some people have said it feels very academic. You have done quite a good job of pulling together all those current literatures all methodologically sound and all that, but you know it is quite difficult to digest as a user in the field. I can see where they are coming from because we’re – we are researchers and we’re also trying to adhere to these WHO standards which require sort of – kind of requirements of certain kinds of language and so on. I think that is probably true of all the evidence coming out of this project, that’s how we kind of make those things more accessible.*” (002, Member of Secretariat of WHO guidance panel on task shifting)

#### Obtaining and maintaining meaningful buy-in

The need to obtain and maintain buy-in in the development of new products was mentioned by one participant, but the concept resonated with what many of the other participants stated.

“*It is not the member state, it’s not WHO, it’s not the NGOs* [non-governmental organisations]*, it takes many to tango. It’s more like carnival, it’s not like a tango. But it’s different at the same time because if you want to overcome those obstacles that exist, this petty politics and things like this, you really have to… persevere, it requires lots of perseverance. You have to talk all the time, you have to go to the right people all the time, you have to convince a higher up, a senior person, and then this person gets distracted and you have to go back and press, and you have to ask your friends to call this person. It’s relentless*.” (007, Staff of WHO not part of Secretariat of guidance panel on task shifting)

As noted in the journal, reflecting on these concepts during the analysis led to thinking about institutionalisation as a type of ‘permanent buy-in’ by an institution. Unless processes of including tools to contextualise guidance at the WHO level or of using these tools at the country level are institutionalised, a change in leadership could result in previous buy-in being lost. Therefore, either continuous communication with new leadership is required to secure meaningful buy-in or institutionalising a process by one group or leader could bypass the future need to obtain buy-in. One participant remarked that the difficult part can be knowing if there is meaningful buy-in or if the buy-in is for a secondary purpose (e.g. advancing other work). This could manifest itself as appearing to have meaningful buy-in at one stage of the process but not at another.

“*My sense is that the panel which had more health systems and policy people on it, was very very supportive… but from the WHO staff people we continued to have this problem that if they come from clinical epidemiology backgrounds, their sense is their usual way of doing by and large can be aloofness, but they recognise that they sometimes still need to have people like me in the room to make it look like they are doing things differently, but I am not convinced at the end of the day that they are committed to doing things differently.*” (001, Health systems and policy analyst)

However, ascertaining this level of information could require immense transparency on the part of the actors involved as it seems unlikely for people to be open about secondary motives. As was noted in the journal, the problem of buy-in could also be difficult to distinguish from a separate problem of lacking resources (e.g. buy-in from one group of people may not secure resources from other groups of people).

#### Ensuring access to resources

Most participants noted the need for human resources, finances and time for different steps in the process of developing the workbook and contextualising the guidance. In addition, several participants mentioned that having people from different organisations involved in the work with WHO also meant there were different agendas, timelines and priorities involved in developing the guidance and the workbook and in implementing the guidance recommendations.

“*So right now, you know, everyone puts all the effort into the front end and then the guidance is there and there is no energy or money to see it implemented….we really need to re-think how we develop critical paths for developing guidance and the guidance development process ends relatively early in that timeline and then we have lots of time and resources left to do all this other stuff. Otherwise the whole exercise is for naught*.” (001, Health systems and policy analyst)

Human resources include the people involved in doing the work. Further attributes of these individuals, which arose from the data, include motivation and knowledge. Motivation to work on a particular topic is necessary when there are competing demands on an individual or on an agency. This can be seen as prioritising specific work. Knowledge can be intrinsic and come from existing knowledge of the individuals involved in the work (e.g. expertise) or can be extrinsic and found through searches for information (e.g. library resources). Finances include salaries or payments for those carrying out the work and funding for traveling and for securing supplies. Time is required for individuals to do the work, and individuals and agencies have timelines for getting the work done. Technology can also support (e.g. find evidence) or be a barrier (e.g. complex or limited access) in advancing the work.

## Discussion

### Principal findings

Three discrete steps were identified in the process of developing the workbook for health systems guidance contextualisation, namely (1) determining the need for and gaining approval to develop the workbook, (2) developing the workbook and (3) implementing the workbook both at the WHO level and at the national/subnational level. Five factors affecting each step in the development of the workbook surfaced as barriers and/or facilitators, namely (1) having well-placed and credible champions, (2) creating and capitalising on opportunities, (3) finding the right language to engage various actors and obtain buy-in, (4) obtaining and maintaining meaningful buy-in, and (5) ensuring access to resources.

Each of these factors was found to be present when proceeding from one step to the next in the process. However, some factors played larger roles at certain points. For example, in developing the workbook, the sub-step of taking on the task relied heavily on champions, language and buy-in, while the sub-step of creating the structure of the workbook relied more on resources. Steps 1 and 2, determining the need for and gaining approval to develop the workbook and developing it, were both successful in that the workbook, along with the OptimizeMNH guidance, was published online. Barriers encountered during these two steps were overcome. However, the implementation of the workbook at the WHO level and at the national/subnational level have encountered many barriers and have yet to occur. The exceptions are Peru and Uganda, where the use of the workbook has been evaluated (separate study).

Several points are noteworthy. Another instance was identified where WHO-led work has not been taken up systematically. The development of the Handbook for Developing Health Systems Guidance [37], while not central to this study, came up sufficiently during the interviews to enable observations to be made about its process of development. The Handbook was supported in its production by WHO, but it was ultimately not published or endorsed by WHO and has not had systematic uptake within WHO. The extent of its use by departments in WHO is therefore unknown. This represents another lost opportunity for advancing the work in health systems strengthening. Instead, as evidenced through the interviews, groups within WHO are trying to distinguish between health systems guidance and clinical guidelines without referring to the work of the taskforce that developed the Handbook. It is unknown at this time if the workbook will have consistent uptake or if future WHO guidance documents will require the incorporation of tools to help with contextualisation.

### Fit within existing literature

As mentioned previously, the opportunity to study the development of the workbook was pragmatic in that events were taking place before there was enough time to conduct a full literature search to identify all the frameworks around the creation of a new knowledge translation tool, especially within similar settings to WHO. Instead, events were noted as they occurred. A critical interpretive synthesis of the literature around contextualisation of guidance (separate study) identified many frameworks and tools for the dissemination and implementation of clinical practice guidelines [38–47]. Many of these tools (e.g. ADAPTE, MAGICapp, EtD) are used at the development, dissemination, adaptation or implementation of guidelines or recommendations at the service or provider levels. Many do not account for the health (e.g. referral systems, supply chain management, regulatory changes for providers) and/or political systems (i.e. institutions, interests, ideas and external factors) changes that may be necessary to support the implementation of guidelines. This was the main reason a new tool was sought to support the implementation of the OptimizeMNH guidance. As opposed to adapting recommendations directly, the workbook addresses how possible policy solutions can be developed to support the implementation of guidance recommendations at the systems level by considering these contextual factors. The study by Gagliardi et al. [48] highlights the importance of context in the implementation of integrated knowledge translation (IKT), or collaboration between researchers and policy-makers. IKT may be seen as a tool in the use of research evidence for decision-making, and each organisation needs to develop its own approach to IKT. Challenges and enablers affecting the success of IKT were similar to those found in this study and included champions, opportunities, organisational endorsement, resistance to change, resources, motivation and time. Further, findings in this study align with some of the concepts from Greenhalgh et al.’s [49] review of diffusion of innovations frameworks, including champions, leadership and vision, enablement of knowledge sharing via internal and external networks, values and goals, power balance, innovation-system fit, dedicated time/resources, and motivation. These frameworks may be especially useful in addressing the dissemination and implementation of workbooks at the WHO and national/subnational levels, or the third step in our process. However, this study extends the findings from these frameworks to the multiple steps in the creation of new tools, not just at the diffusion or implementation stage. Each step may be seen as its own process, which is helpful for those planning to undertake a similar task. This study also reinforces the use of qualitative methods to examine the process and context of developing a new knowledge translation tool.

### Strengths and limitations of the study

The main strengths of this paper revolve around the choice of methods. Following a qualitative case study method, including the use of multiple sources of data such as interviews, documents and a reflexive journal, allowed for an in-depth look at the process of developing the workbook. The role of participant-observer added to the richness of the findings in that EA and JL had first-hand accounts of the process. Member checking and peer debriefing helped strengthen the analysis and increased rigor. Finally, the reflexive journal helped identify the investigator’s biases (e.g. beliefs that health systems guidance and the contextualisation of guidance hold promise in strengthening health systems) and account for them to mitigate their influence, such as by asking for both positive and negative feedback on the workbook during the interviews.

There is one main weakness of this study, which is the potential for recall bias with retrospective interviews (ranging from 6 months to 4 years). However, having multiple interviewees and triangulating information with documents helped decrease the impact of this recall bias. One main challenge with the case study methodology is that findings cannot be generalised from a single case to other cases, as the context of the work done within and alongside an international organisation such as WHO may be unique. Therefore, readers would need to consider their own contexts before applying these findings to their settings. A limitation includes that testing the workbook was not conducted through this study. This work was instead conducted through a separate study evaluating the use of the workbook in Peru and Uganda (in review).

### Implications for policy and practice

There are three main implications for policy and practice that were found through this study. First, lack of implementation of guideline recommendations is still a significant barrier to improving care, and there has been a call to increase attention to implementation strategies within documents developed by WHO [11]. This study provides some clear and direct steps that can be used to develop new tools for WHO and similar organisations. Practically, the workbook was developed to be generic enough to use for any policy topic, yet incorporates examples drawn from the OptimizeMNH guidance. Subsequent versions of the workbook would not take as long to develop as it would simply rely on drawing examples for other particular guidance documents. Second, as was seen in this study, and in the case of the Handbook, a plan for dissemination and implementation of the tools themselves needs to be addressed in their preparation. Not having a plan for implementation can severely limit the use and therefore the usefulness of these labour- and resource-intensive products. Third, many considerations were listed as barriers to the implementation of workbooks at the WHO and national/subnational levels. These are all areas that could be addressed in practice so that guidance recommendations have a better chance of being implemented and can have a positive impact on the health of populations. Furthermore, the role of various stakeholders, such as guideline development at the WHO level versus a more tailored approach to guideline development at the regional or national level, is an area that needs further elaboration to determine the right balance of resource use and context-tailored recommendations. This is especially important in light of equity considerations.

### Implications for research

First, using qualitative methods to study processes of tool development can help not only to describe the process but also its context to tease out facilitators and barriers in these processes. Second, the use of the workbook has been evaluated in two countries, Peru and Uganda (in review). However, in order to improve guidance contextualisation processes at the national/subnational levels, further evaluations of the workbook for various topics, and in different contexts, will be needed to help refine the language and structure to make the workbook more user-friendly and therefore more useful.

## Conclusions

The key steps and critical factors found through this study would help in the planning of other tools at WHO. Additionally, a plan for dissemination and implementation needs to be addressed during the preparation of these tools. As this study highlights, there are many barriers in the implementation of this workbook at the WHO level and at the national/subnational level, which can be addressed in practice. This study also provides insights into improving the workbook from the perspective of international guidance developers.

## Additional files

Additional file 1: Documents reviewed for the study. (DOCX 18 kb)

Additional file 2: Critical factors influencing each step of the process of workbook development. (DOCX 19 kb)

## Abbreviations

IKT: integrated knowledge translation
LMICs: low- and middle-income countries
OptimizeMNH: Optimizing health worker roles to increase access to and utilization of key interventions to improve maternal and newborn health
