# Supplementary material for: Developing a workbook to support the contextualisation of global health systems guidance: a case study identifying steps and critical factors for success in this process at WHO
Source: Health Res Policy Syst. 2018 Mar 2;16:19. doi: 10.1186/s12961-018-0297-x (PMC5833055; doi:10.1186/s12961-018-0297-x)
Supplement: Supplementary file 1 — Documents reviewed for the study. (DOCX 18 kb) [file 12961_2018_297_MOESM1_ESM.docx]

Additional file 1. Documents reviewed for the study

| **Date** | **Document Title** | **Author** | **Document Type** | **Source** |
| --- | --- | --- | --- | --- |
| 2003  March | Guidelines for WHO guidelines | Global Programme on Evidence for Health Policy | Document | Internet search |
| 2004;  2011 (Updated) | Ministerial Summit on Health Research, Mexico City, Mexico - November 16-20, 2004 | WHO | Documents | Interviewee |
| 2004 | Informed choices for attaining the millennium development goals: towards an international cooperative agenda for health-systems research | Task Force on Health Systems Research | Journal article | Interviewee |
| 2004 | Overcoming health-systems constraints to achieve the millennium development goals | Travis et al | Journal article | Interviewee |
| 2007 | Use of evidence in WHO recommendations | Oxman et al | Journal article | Personal email |
| 2007 | WHO signals strong commitment to evidence | No author listed | Journal article | Personal email |
| 2007 | Everybody’s Business. Strengthening health systems to improve health outcomes. WHO’s framework for action. | WHO | Document | Proposal to Rockefeller Foundation. 2009. |
| 2008 | Task shifting: rational redistribution of tasks among health workforce teams. Global recommendations and guidelines | WHO | Document | Personal email |
| 2009 | Systems thinking for health systems strengthening | Alliance for Health Policy and Systems Research (AHPSR), WHO | Document | Bosch-Capblanch X. Development of a protocol to elaborate health system guidelines: Pre-proposal. Swiss Centre for International Health; 2010. |
| 2009 | Proposal to Rockefeller Foundation | No author listed | Document | Multiple interviewees |
| 2009 | The use of research evidence in two international organizations’ recommendations about health systems | Hoffman et al | Journal article | Personal email |
| 2009 | SUPPORT Tools for evidence-informed health policymaking (STP) 1: What is evidence-informed policymaking? | Oxman et al | Journal article | Personal email |
| 2009 | SUPPORT Tools for evidence-informed health Policymaking (STP) 4: Using research evidence to clarify a problem | Lavis et al | Journal article | Personal email |
| 2009 | SUPPORT Tools for evidence-informed health Policymaking (STP) 5: Using research evidence to frame options to address a problem | Lavis et al | Journal article | Personal email |
| 2009 | SUPPORT Tools for Evidence-informed Policymaking in health 6: Using research evidence to address how an option will be implemented | Fretheim et al | Journal article | Personal email |
| 2009 | SUPPORT Tools for evidence-informed health Policymaking (STP) 9: Assessing the applicability of the findings of a systematic review | Lavis et al | Journal article | Personal email |
| 2009 | SUPPORT Tools for evidence-informed health Policymaking (STP) 10: Taking equity into consideration when assessing the findings of a systematic review | Oxman et al | Journal article | Personal email |
| 2009 | SUPPORT tools for evidence-informed policymaking in health 11: Finding and using evidence about local conditions | Lewin et al | Journal article | Personal email |
| 2009 | SUPPORT Tools for evidence-informed health Policymaking (STP) 12: Finding and using research evidence about resource use and costs | Oxman et al | Journal article | Personal email |
| 2009 | SUPPORT Tools for evidence-informed health Policymaking (STP) 13: Preparing and using policy briefs to support evidence-informed policymaking | Lavis et al | Journal article | Personal email |
| 2009 | SUPPORT Tools for evidence-informed health Policymaking (STP) 14: Organising and using policy dialogues to support evidence-informed policymaking | Lavis et al | Journal article | Personal email |
| 2009 | SUPPORT Tools for evidence-informed health Policymaking (STP) 15: Engaging the public in evidence-informed policymaking | Oxman et al | Journal article | Personal email |
| 2009 | SUPPORT Tools for evidence-informed health Policymaking (STP) 16: Using research evidence in balancing the pros and cons of policies | Oxman et al | Journal article | Personal email |
| 2009 | SUPPORT Tools for evidence-informed health Policymaking (STP) 17: Dealing with insufficient research evidence | Oxman et al | Journal article | Personal email |
| 2009 | SUPPORT Tools for Evidence-informed Policymaking in health 18: Planning monitoring and evaluation of policies | Fretheim et al | Journal article | Personal email |
| 2010 | The global health system: actors, norms, and expectations in transition | Szlezak et al | Journal article | Interviewee |
| 2010 | The global health system: strengthening national health systems as the next step for global progress | Frenk | Journal article | Interviewee |
| 2010 | The global health system: linking knowledge with action-learning from malaria | Keusch et al | Journal article | Interviewee |
| 2010 | The global health system: lessons for a stronger institutional framework | Moon et al | Journal article | Interviewee |
| 2010  Feb | Development of a protocol to  elaborate health system guidelines: Pre-proposal | Bosch-Capblanch | Document | Interviewee |
| 2010  March | WHO handbook for guideline development | WHO | Document | Personal email |
| 2010  April 15-16 | Task force on guidelines for health systems strengthening - draft agenda for meeting | No author listed | Document | Interviewee |
| 2010 November | Optimizing the delivery of key interventions to attain MDGs 4 and 5: Background document for the First Expert ‘Scoping’ Meeting to Develop WHO Recommendations to Optimize Health Workers’ Roles to Improve Maternal and Newborn Health in Geneva, 6-8 December 2010 | WHO: Department of Reproductive Health and Research | Document | Personal email |
| 2010 December | Optimizing the delivery of key interventions to attain MDGs 4 & 5: Draft agenda - December 2010 scoping meeting - Centre International de Conférence Genève (CICG), Geneva, Switzerland 6-8 December 2010 | WHO | Document | Personal email |
| 2010 December | Optimizing the delivery of key interventions to attain MDGs 4 & 5: background and approach | Oladapo | Presentation | Personal email |
| 2010 December | Optimizing the delivery of key interventions to attain MDGs 4 & 5: meeting objectives. | Gülmezoglu | Presentation | Personal email |
| 2010  December 16 | Notes from the Technical Consultation on Optimizing the Delivery of Key Healthcare Interventions to attain MDGs 4 & 5: 6-8 December 2010, Geneva, Switzerland | No author listed | Document | Personal email |
| 2011  May 31-June 1 | The guideline development process: WHO recommendations for optimizing the delivery of key interventions to attain MDG 4 and 5 through task shifting | No author listed | Presentation | Personal email |
| 2011  May 31-June 1 | Optimizing the delivery of key interventions to atain MDGs 4 & 5 through task-shifting: Background and objectives | Gülmezoglu | Presentation | Personal email |
| 2011 | Handbook for supporting the development of health system guidance | Bosch-Capblanch | Document | Multiple interviewees |
| 2011 (Updated) | SURE guides for preparing and using evidence-based policy briefs | WHO / SURE | Documents | Interviewee |
| 2012 | Safe abortion: technical and policy guidance for health systems | WHO, Department of Reproductive Health and Research | Document | Personal email |
| 2012 | Guidance for evidence-informed policies about health systems: rationale for and challenges of guidance development. | Bosch-Capblanch et al | Journal article | Personal email |
| 2012 | Guidance for evidence informed policies about health systems: linking guidance development to policy development | Lavis et al | Journal article | Personal email |
| 2012 | Guidance for evidence informed policies about health systems: assessing how much confidence to place in the research evidence | Lewin et al | Journal article | Personal email |
| 2012 | Better guidance is welcome, but without blinders | Peters & Bennet | Journal article | Interviewee |
| 2012  April 16-19 | Optimizing the delivery of key interventions to attain MDGs 4 & 5: Draft agenda - Geneva, Switzerland 16-19 April 2012 | WHO | Document | Personal email |
| 2012  April 16-19 | Optimizing the delivery of key interventions to attain MDGs 4 & 5 through task-shifting | Gülmezoglu | Presentation | Personal email |
| 2012  April 19 | Supporting the contextualization of the guideline | Lavis | Presentation | Personal email |
| 2012  June 7 | Supporting the contextualization of the Optimize4MNH Guidance at the national level | Lavis | Presentation | Meeting |
| 2012 | OptimizeMNH: Optimizing health worker roles to improve access to key maternal and newborn health interventions through task shifting | WHO | Document | Interviewee |
| 2012 | Annex 8. Contextualizing the guidelines - workbook | WHO | Document | Principal investigator, online search |
| 2012, Mar 3 –  2013, Nov 19 | Reflexive Journal I (Workbook) | Principal investigator | Reflexive Journal –  195 pgs, | Principal investigator |
| 2013, Nov 20 –  2014, Jun | Reflexive Journal II (Workbook) | Principal investigator | Reflexive Journal –  127 pgs | Principal investigator |
| 2014, June 5 –  2015, Jan 23 (ongoing) | Reflexive Journal III (Workbook) | Principal investigator | Reflexive Journal –  93 pgs | Principal investigator |
| 2011-2014 |  | Emails, 2011-2014 | Personal correspondence | Personal emails, some forwarded through interviewees |
