# Supplementary material for: Developing a workbook to support the contextualisation of global health systems guidance: a case study identifying steps and critical factors for success in this process at WHO
Source: Health Res Policy Syst. 2018 Mar 2;16:19. doi: 10.1186/s12961-018-0297-x (PMC5833055; doi:10.1186/s12961-018-0297-x)
Supplement: Supplementary file 2 — Critical factors influencing each step of the process of workbook development. (DOCX 19 kb) [file 12961_2018_297_MOESM2_ESM.docx]

Additional file 2. Critical factors influencing each step of the process of workbook development

| **Themes** | **Barrier(s)** | **Facilitator(s)** | **Example(s)** |
| --- | --- | --- | --- |
| **Step 1 – Determining the need for and gaining approval to develop the workbook** – WHO guidance panel on task shifting | | | |
| Champions |  |  | The head of the Secretariat of the WHO guidance panel on task shifting and an expert on health systems policy acted as champions by creating and capitalizing on opportunities, by helping move forward the idea for a workbook on contextualizing guidance recommendations, and by building buy-in. |
| Opportunities |  |  | An opportunity was created by the head of the Secretariat of the WHO guidance panel on task shifting by asking an expert on health systems policy to join and co-chair a panel meeting. The expert capitalized on several opportunities to advance the work and build buy-in. A further opportunity was created in that another member of the Secretariat of the guidance panel was also involved in work that was concurrently being carried out by WHO on developing health systems guidance. |
| Language |  |  | Because this work was new, people were unfamiliar with it, which created a challenge. Using common terminology helped build understanding and persuade those involved in the process about the need for this work. |
| Buy-in |  |  | The Secretariat of the WHO guidance panel on task shifting, the panel members of the WHO guidance panel on task shifting, and others at WHO bought-in to the need for this work. Even though some panel members did not have a background in health systems, which created a challenge, the champions found the right language to engage the panel members and obtain buy-in. |
| Resources |  |  | Human resources, time and funding for carrying out the work of the WHO guidance panel on task shifting were all secured. |
| **Step 2- Developing the workbook** – sub-steps included: taking on the task, creating the structure of the workbook, operationalizing its components, undergoing approval processes and editing it | | | |
| Champions |  |  | The health systems policy expert acted as champion by bringing the idea up to someone who could take on this work, by acting in a supporting role throughout the development of the workbook, and by promoting the use of the workbook. A participant-observer acted as champion by developing the workbook and iteratively revising it throughout the development process, and by promoting the use of the workbook. |
| Opportunities |  |  | The health systems policy expert presented the opportunity to develop the workbook to the participant-observer taking on this work |
| Language |  |  | The health systems policy expert presented the idea of developing the workbook as a win-win situation to the participant-observer who took on the work. Operationalizing the workbook was important to explain how to use the workbook, especially for those without training in health policy. One challenge during the approval process was that the language of the workbook had to conform to the standards of the WHO Guideline Review Committee (GRC), therefore changes to the workbook were made (e.g. a 2-page summary was developed). |
| Buy-in |  |  | Buy-in was obtained from the participant-observer to take on the development of the workbook and by the WHO GRC to approve the inclusion of the workbook. Buy-in was maintained through the development and approval processes from the Secretariat of the WHO guidance panel on task shifting, which also helped shape the design of the workbook. |
| Resources |  |  | Human resources, dedicated time, motivation, knowledge and technology were used in developing the workbook. Having no previous example of a workbook to contextualize health systems guidance created a challenge, but this was overcome by using knowledge from prior work done in the field. Researching and sharing information was done with the use of technology. Having multiple agencies involved in this work created some challenges with competing timelines and agendas but it also helped focus attention on the development of the workbook. The workbook was published online, but the final execution of the proposed edits did not occur, which could have been due to a combination of not having true buy-in by the Secretariat of the WHO guidance panel on task shifting or due to a lack of time and other resources. |
| **Step 3a – WHO level implementation of the workbook:** Institutionalizing the development of workbooks for guidance documents at WHO and disseminating and supporting the use of such workbooks at the national level | | | |
| Champions |  |  | Champions exist for this work and have been trying to gain traction to incorporate workbooks for contextualizing health systems guidance into guidance development processes at WHO, but, thus far, the necessary buy-in from those with decision-making power has not been gained. In addition, a Secretariat member who had acted as champion at earlier stages of the development of the workbook did not act as champion for the full institutionalization of the workbook idea or the dissemination of this workbook. |
| Opportunities |  |  | There is a need for support in contextualizing health systems guidance recommendations at the national/subnational level, and this workbook is meant to address this need. However, there have been several missed opportunities including that this process has not been institutionalized at the WHO level, WHO has not been involved in evaluating the use of the workbook at the national/subnational level, and prior work on developing health systems guidance was also not institutionalized at WHO. |
| Language |  |  | Language could act as a barrier to institutionalizing the work within WHO or at the national or subnational levels if the language is not easy for end-users to understand (e.g., too detailed, jargon, English-specific concepts). |
| Buy-in |  |  | There are rival theories as to whether differing viewpoints (clinical epidemiology vs. health systems and policy) have acted as barriers in institutionalizing workbooks to accompany health systems guidance or whether there is understanding and agreement with the importance of this work by those who have decision-making power at WHO and instead there are political or personal issues that stand in the way. |
| Resources |  |  | Resources at the WHO level were put into developing and disseminating the OptimizeMNH guidance, but these resources were not used in parallel to disseminate or support the use of the workbook at the national or subnational level. |
| **Step 3b – National/subnational level implementation of the workbook:** using, evaluating and institutionalizing the use of the workbook to develop health systems policy | | | |
| Champions |  | Peru and Uganda | It can be challenging to finding champions within countries to move this work forward. However, Peru and Uganda have found champions who, along with support from those who developed the workbook, have agreed to use the workbook to develop health systems policy and to allow for the evaluation of using the workbook. |
| Opportunities |  | Peru and Uganda | Dissemination of the OptimizeMNH guideline and the workbook occurred at the WHO African Regional office (WHO-AFRO) Regional Consultation on Optimizing Health Workers’ Roles to Improve Access to Key MNH Interventions Through Task-shifting in Addis Ababa in 2012. However, there were some missed opportunities at this event to offer support to countries to evaluate the use of the workbook in their countries. Opportunities were created for two countries through different channels. A potential opportunity exists for this process to identify gaps in knowledge, which can, in turn, inform the research process itself. |
| Language |  |  | Preliminary feedback suggests that the workbook may be helpful, as it does provide a systematic approach to contextualizing health systems guidance, but it is too long and detailed overall, and people using the workbook may need some training in health policy, or support, in order to understand the complexities of the components of the workbook. Field testing will be the best way to evaluate the workbook and ensure it is understandable and relevant to the users. |
| Buy-in |  | Peru and Uganda | While some countries have asked WHO for support in contextualizing guidelines, some regions or countries do not place much importance on evidence and guidelines. Several participants noted that there may be a need for a culture shift towards developing health systems policy and evidence-informed policymaking in these regions or countries. In addition, it was noted that buy-in is required from policymakers but also from those who are responsible for implementing policies at the national or subnational level. In Peru and Uganda, buy-in by those champions at the country level was secured as they already placed importance in contextualizing health systems guidance for their settings. The workbook may be helpful in building ownership to improve buy-in by including processes for stakeholder and public engagement. |
| Resources |  | Peru and Uganda | Resources for evidence-informed policymaking are often lacking at the national or subnational level. Multiple funding agencies and priority-setting processes within most countries can make carrying out this work difficult. Many countries have little capacity for research or for embedding this work into guidance development or policymaking processes and they do not have adequate resources to see implementation through. There is a need to build a health system evidence base over time to know what works within which contexts, and even though technology can be an asset by allowing more people to have access to more information, in many settings, people lack access to technology. Human resources and some financial resources helped support the evaluation of the use of the workbook in Peru and Uganda. |
